# Habitat quality effects on the abundance of a coral‐dwelling fish across spatial scales

**DOI:** 10.1002/ece3.70322

**Published:** 2024-09-22

**Authors:** Hana Fahim, Taylor Naaykens, Cassidy C. D'Aloia

**Affiliations:** ^1^ Department of Biology University of Toronto Mississauga Mississauga Ontario Canada; ^2^ Department of Ecology and Evolutionary Biology University of Toronto Toronto Ontario Canada

**Keywords:** coral reef, cryptobenthic fish, marine symbiosis, microhabitat specialist, reef degradation

## Abstract

Microhabitat associated fishes are expected to be negatively affected by coral reef degradation, given that many species are coral dwellers. However, the factors underlying this negative impact and the spatial scale(s) at which it occurs are poorly understood. We explored how habitat quality metrics and host preferences influence fish abundance across multiple spatial scales, using the functionally important cleaner fish *Elacatinus evelynae* as a study species. We surveyed fish at 10 sites in Curaçao that varied in coral cover and health. At the microhabitat scale, we found that *E*. *evelynae* group size increases on large, healthy corals and on some coral host species, namely *Montastraea cavernosa*. We also found that, although *E*. *evelynae* can occupy at least 10 coral host species, it selectively inhabits just three corals: *M*. *cavernosa*, *Colpophyllia natans*, and *Diploria labrynthiformis*. Scaling up to explore goby abundance along 30‐m transects, we did not find a clear relationship between live coral cover and goby abundance. However, goby abundance was substantially higher at one location with elevated coral cover and a high relative abundance of *E*. *evelynae* host species. Collectively, these results confirm that *E*. *evelynae* abundance is impacted by reef health. They also indicate that the species' long‐term persistence may depend on both the maintenance of healthy coral hosts and the gobies' plasticity in host preferences on changing reefscapes. Cryptobenthic fishes such as *E*. *evelynae* play a vital role in the ecosystem and understanding drivers of their abundance is important as reefs face increased degradation.

## Graphical abstract

Coral‐dwelling fishes are expected to be negatively affected by reef degradation, but the factors underlying this relationship are poorly understood. We showed that the functionally important cleaner fish *Elacatinus evelynae* is affected by habitat quality metrics across spatial scales, requiring large, healthy coral heads and preferring a subset of host species. Our results indicate *E*. *evelynae* may be vulnerable to extirpation at very low levels of coral cover.

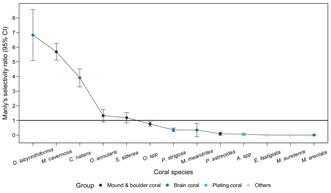

## 1 INTRODUCTION

Coral reefs, an important habitat for more than 25% of marine species and almost a billion people, have declined by at least 50% globally in the past 50 years (Eddy et al., 2021; Fisher et al., 2015; Sing Wong et al., 2022). Reef degradation entails a loss in corals, which, in turn, decreases habitat heterogeneity and structural complexity (Pratchett et al., 2014). Degradation is driven by climate‐induced bleaching (Bove et al., 2022; Hughes et al., 2018), disease (Alvarez‐Filip et al., 2019), and local pressures such as overfishing, tourism, pollution, and eutrophication (Andrello et al., 2022). The ongoing loss of live coral presents a major challenge to fishes that associate closely with reefs during at least part of their life cycle (Alvarez‐Filip et al., 2015; Budd et al., 2024; Jones et al., 2004; Wilson et al., 2006).

Cryptobenthic fishes—small fishes that maintain a strong association with the benthos (Brandl et al., 2018; Depczynski & Bellwood, 2003)—are often overlooked in studies on the response of fishes to reef degradation (Ahmadia et al., 2012; Brandl et al., 2019). These species play an important functional role through high biological productivity and transferring energy to higher trophic levels (Ahmadia et al., 2012; Brandl et al., 2019; Depczynski & Bellwood, 2003). Because many cryptobenthic fishes rely on corals for habitat, they may be especially susceptible to the impacts of reef degradation via coral loss and shrinkage. For example, Froehlich et al. (2021) found that *Gobiodon* gobies, a genus of cryptobenthic fishes that inhabit *Acropora* corals, experienced strong declines in their abundance following multiple disturbance events that led to the loss of some coral hosts and a decrease in average coral host size.

Among coral dwellers, species vary in their degree of microhabitat specialization, i.e., in the number of coral taxa inhabited. Specialized fishes that inhabit few coral species are more strongly affected by coral loss than generalists (Alvarez‐Filip et al., 2015; Kochan et al., 2023; Munday, 2004; Wilson et al., 2008) and, consequently, may experience elevated extirpation risks, especially in an environment of ongoing reef degradation (Bonin, 2012). Therefore, it is critical to assess species' host preferences and their degree of microhabitat specialization. It is also important to elucidate the spatial scale(s) at which degradation affects coral‐dwelling fishes. For example, at the smallest scale, the occupancy and group size of these fishes can be strongly impacted by features of their host, including coral colony size (Chase & Hoogenboom, 2019; Froehlich et al., 2021; Thompson et al., 2007) and coral health (Budd et al., 2024; Coker et al., 2012; Smallhorn‐West et al., 2017). These host‐level effects on fish abundance could also scale up to larger reef areas, such that fish abundance correlates with larger‐scale factors, such as site‐level coral community composition or average live coral cover (Boström‐Einarsson et al., 2013; Russ et al., 2021).

One cryptobenthic fish that is well suited for exploring the relationship between microhabitat degradation and fish abundance is *Elacatinus evelynae*, the sharknose goby. Distributed throughout much of the Caribbean (Colin, 2010), *E*. *evelynae* is well‐studied as a cleaner fish (Colin, 2010; White et al., 2007; Whiteman & Côté, 2002), whose abundance is partly explained by local client density and their associated parasites (Cheney & Côté, 2003). Although commonly described as living alone or in pairs (Harding et al., 2003; Whiteman & Côté, 2003), individuals can also form large groups in parts of their range (White et al., 2007; Whittey et al., 2021; Wilson & Osenberg, 2002; Ziebell et al., 2023). *Elacatinus evelynae* has been reported to occupy multiple hosts throughout its broad geographic range, including brain, mound, boulder, and plating corals. In St. Croix, they can even host switch into sponges, potentially due to low interspecific competition with sponge‐dwelling *Elacatinus* species (White et al., 2007). Thus, the species' microhabitat associations, and the ecological drivers of those associations, appear to differ regionally. To date, there has been limited research into the effects of habitat quality on *E*. *evelynae's* distribution and abundance. Because the species is a coral dweller, habitat quality metrics related to corals are applicable, and can include both individual host characteristics (e.g., species, size, and health) and live coral cover as a proxy for the health of the local reef. In one study, Cheney and Côté (2003) found no relationship between coral cover and *E*. *evelynae* density, but that study estimated coral cover of the overall reef and not coral cover of the transects where goby density was measured.

In this study, we quantified the distribution and abundance of *E*. *evelynae* in Curaçao and asked the research question: how do habitat quality attributes affect *E*. *evelynae's* abundance and at what spatial scale(s)? We used underwater survey data and a multi‐scale approach to identify the effect of habitat quality on *E*. *evelynae's* abundance at both the microhabitat and 30‐m transect scales, leveraging variation in reef health across Curaçao. Specifically, the objectives of this study were to: (1) determine which coral host species *E*. *evelynae* preferentially associates with; (2) assess the effect of coral host features (e.g., species identity, size, and health) on *E*. *evelynae's* group size at the microhabitat scale; and (3) assess whether coral cover is associated with *E*. *evelynae's* abundance at the 30‐m transect scale.

## 2 METHODS AND MATERIALS

### 2.1 Study sites

**FIGURE 1:**
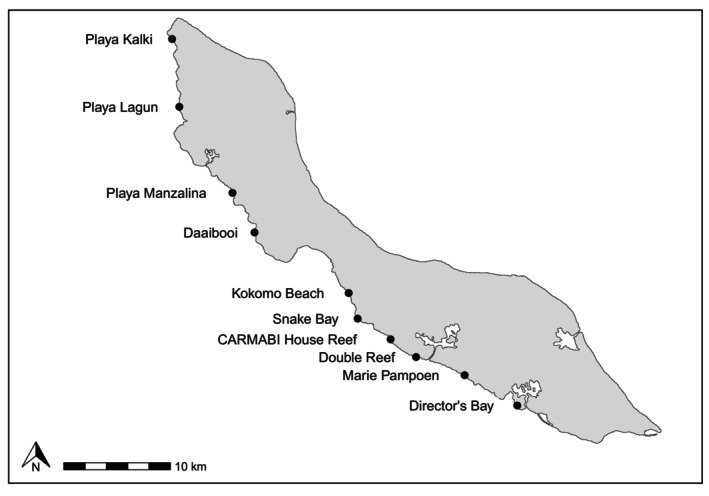
Map of the 10 surveyed sites in Curaçao.

Curaçao's fringing reefs typically exhibit a gradual slope and then a steep drop‐off, extending along the coast of the island (Bak, 1975; Böhm & Hoeksema, 2017; Sandin et al., 2022). In recent decades, Curaçao has experienced limited large‐scale disturbances, including storms, bleaching events, and coral disease (Sandin et al., 2022). However, coral cover has declined substantially over the past few decades, and the island has experienced high local threats to the reef, including coastal development, pollution, and overfishing (Burke et al., 2011; Vermeij, 2012). We surveyed 10 sites spread along the coast on the leeward side of the island (Figure 1). Sites were selected to cover a large geographic area and capture variation in reef degradation based on previous reports of coral cover and human impacts (CARMABI and Waitt Institute, 2017).

### 2.2 Benthic and goby surveys

To quantify benthic cover and estimate *Elacatinus evelynae* abundance and microhabitat characteristics, SCUBA‐based surveys were conducted during June and July 2022. At each site, 30 m long transects were conducted parallel to shore at depths of 5.0, 7.5, and 10.0 m (*n* = 2 transects per depth for most sites). Three transects per depth were surveyed at CARMABI House Reef. At Playa Lagun, only one transect was surveyed at 5.0 and 7.5 m because the cove was too narrow at 5.0 m and the seafloor was entirely sand at 7.5 m. This resulted in 61 total transects across the 10 sites. For benthic cover estimation, we took a video of the transect using a GoPro HERO9, swimming at a steady pace approximately 0.5 m above the seafloor.

To estimate the abundance of gobies and their microhabitats, we followed the same transect lines used for the benthic cover estimation and applied a 3.0 m wide belt transect. All potential microhabitats, including live hard coral, sponges, and rocks, were checked for the presence of *E*. *evelynae*. At each microhabitat where at least one *E*. *evelynae* was present, we counted the total number of individuals, representing group size. We also summed all *E*. *evelynae* recorded on a transect to obtain the abundance per 30‐m transect.

At each microhabitat, we also measured its characteristics including species, size, and, for coral hosts only, health. We identified the host to the species level, except for some species of the coral genus *Orbicella*, namely, *O*. *faveolata* and *O*. *franksi*, and *Agaricia* species, including *A*. *agaricites* and *A*. *lamarcki*, which were identified to the genus level because they are challenging to distinguish based on morphology. For microhabitat size, we measured height, length, and width (cm) for corals and calculated the average diameter as a three‐dimensional size metric (Froehlich et al., 2023; Kuwamura et al., 1994). For sponges, we measured maximum tube height as a proxy for overall size (sensu D'Aloia et al., 2011, Naaykens et al., 2024). For corals, health was also recorded based on visual estimates of the approximate percentage of live tissue and coded as an ordinal categorical variable with three levels: degraded (<50% live tissue), intermediate (50–90% live tissue), and healthy (>90% live tissue). Coral health was not estimated at CARMABI House Reef or Kokomo Beach. For consistency, one diver did all fish counts and the other did all microhabitat measurements.

### 2.3 Estimation of benthic cover

Images were extracted from the video transects using FFmpeg (Newmarch, 2017). Per transect, 20 non‐overlapping images were extracted at equal time intervals, resulting in a total of 1220 images across all transects. The area captured by the images varied since the videos differed in the exact height above the seafloor (e.g., due to sudden drops in depth where there were grooves in the reef). To quantify photo size variability and select an appropriate size range, we took a random sample of 100 images and measured their length using ImageJ (Schneider et al., 2012), using the transect tape as a size reference. The image sample had a length range of 8.09–80.13 cm (*mean* = 32.98; *SD* = 14.03) and we set the acceptable image length range to 10–60 cm. Both cut‐offs were within two SD of the mean. All remaining images (*n* = 1120) were visually assessed using the transect tape visible in the images to measure length, and any images outside the acceptable range were modified. Images with a length larger than the acceptable range were cropped to the maximum length of 60 cm. Images with a length lower than 10 cm were excluded and replaced by resampling the closest frame within the acceptable range.

Images were uploaded to CoralNet (Williams et al., 2019) for benthic annotation. Simple random sampling with 25 points per image was used to estimate benthic cover. The microhabitat beneath each point was recorded, and we manually checked all CoralNet annotations. Stony corals were identified to species level (or genus, where species identification was not possible). Other benthic categories included sand, rock, rubble, invertebrates (including sponges, anemones, and soft coral), algae (turf algae and crustose coralline algae could not be reliably distinguished due to image quality), and others (including trash, metal pipes, etc.). The number of points in each category was summed and converted to a percent cover. The percent cover was then averaged across all images per transect to determine mean benthic cover for each transect.

### 2.4 Statistical analysis

Data were processed, analyzed, and visualized in RStudio version 2023.09.0 using the R programming language version 4.3.1 (R Core Team, 2023).

#### 2.4.1 *Elacatinus evelynae* association with coral host species

To identify which species of corals *E*. *evelynae* strongly associates with, we calculated Manly's selectivity index for all species of corals identified in our study sites (Manly et al., 2002). The index is a ratio of the proportion of used habitat versus the proportion of available habitat and is calculated as:

(1) $${\hat{w}}_{i}=\frac{o_{i}}{\pi_{i}}$$

where $o_{i}$ is the proportion of gobies observed on coral species i summed across all transects and $\pi_{i}$ is the percent cover of coral species i averaged across all transects. A ${\hat{w}}_{i}$ value of 1 means that *E*. *evelynae* exhibits no preference for a given coral species, a value above 1 indicates a preference for that coral, and a value below 1 indicates an aversion to that coral. We calculated the index with 95% confidence intervals using the “widesI” function in the *adehabitatHS* package (Calenge & Basille, 2023).

#### 2.4.2 Effect of coral microhabitat features on *E*. *evelynae* group size

To explore how microhabitat features influenced goby group size, i.e., the total number of gobies on a single host, we used a generalized linear mixed model with a zero‐truncated negative binomial distribution. The model focused on *E*. *evelynae* living on corals since corals were the predominant microhabitat (*n* = 459 observations). Non‐coral microhabitats had small sample sizes (*n* = 8 observations on sponges; *n* = 11 observations on rocks) and may only serve as temporary stopover habitat. The coral host *Agaricia* spp. (*n* = 4 observations) was also excluded due to difficulty in obtaining three‐dimensional size measurements because of its plating nature. Host characteristics, including host species, health, and depth, were included as fixed effects, average diameter was included as a continuous covariate, and site was included as a random effect. Models were run using the “glmmTMB” function in the *glmmTMB* package (Brooks et al., 2023). The negative binomial distribution was selected because the data exhibited overdispersion, tested using the *performance* package (Lüdecke et al., 2023). A zero‐truncated model was used because the dependent variable, group size, did not include zeros since we only measured microhabitats where at least one goby was present. Model selection was performed using backward stepwise selection based on Akaike's Information Criterion (AIC) with a threshold of ΔAIC >2. The Wald Chi‐squared Test in the *car* package (Fox et al., 2023) was used to assess the statistical significance of fixed effects and the marginal *R* ^2^ was calculated using the *performance* package. Model assumptions were assessed using the *DHARMa* package (Hartig & Lohse, 2022) by visually inspecting residuals. For the categorical (host species) and ordinal (health) independent variables, pairwise comparisons of different levels with a Tukey adjustment were conducted using the “emmeans” function in the *emmeans* package (Lenth et al., 2023).

To assess whether there were significant interactions between variables, we ran additional zero‐truncated negative binomial models using only observations from the preferred coral hosts. Here, we define preferred hosts as species with selectivity indices exceeding 1 (see section 2.4.1 for details). We could not test for interactions using the full data set because the models would not converge. We tested for health × host species and average diameter × host species interactions and assessed significance and model fit as described above. We explored pairwise interactions with the continuous average diameter covariate using the “emtrends” function in the *emmeans* package (Lenth et al., 2023).

#### 2.4.3 Effect of coral cover on *E*. *evelynae* abundance at the 30‐m transect scale

To assess the relationship between coral cover and *E*. *evelynae*'s abundance at the 30‐m transect scale, we used a nonlinear model with a saturating density‐dependent curve that follows the Beverton‐Holt stock‐recruitment curve. This is a zero‐intercept model, which means that when coral cover is zero, i.e., in non‐reef habitat, *E*. *evelynae*'s abundance should also be zero. This model is biologically appropriate as *E*. *evelynae* populations in Curaçao depend on coral habitat (i.e., it is bounded at zero). The Beverton‐Holt is also appropriate because there should be some upper bound of goby abundance on a transect (i.e., goby abundance cannot increase infinitely, and should eventually reach a maximum value based on available coral cover and the characteristics of individual hosts). We used the following parameterization of the Beverton‐Holt function:

(2) $$E\left[R|S\right]=\frac{\mathrm{aS}}{1+\mathrm{bS}}$$

where $E\left[R|S\right]$ is the expected value of R given S (Beverton & Holt, 1957; Ogle, 2016). Here, R is goby abundance and S is coral cover. The density‐independent parameter *a* reflects the slope near *S* = 0 and the density‐dependent parameter *b* influences the asymptote of the curve (Ogle, 2016; Quinn II & Deriso, 1999).

For these models, the unit of observation was a 30‐m transect, i.e., we used the total goby abundance summed across all corals on a given transect as the dependent variable. Three transects, two in Playa Lagun and one in Daaibooi, had zero estimated coral cover. Since these transects did not comprise coral habitat, they were removed from this analysis. We compared two models using different measurements of coral cover: (1) total coral cover and (2) goby‐associated coral cover, i.e., cover of all coral species the gobies were observed on in Curaçao (see Table S1). We found starting values for the parameters a and b (Equation 2) using the “srStarts” function from the *FSA* package (see Ogle et al., 2023 for details on estimating start values) and then obtained final parameter estimates by running the nonlinear model in the “nls” function (Nonlinear Least Squares) from the *stats* package. Model significance was tested by calculating 95% confidence intervals using the “nlsBoot” function from the *nlstools* package (Baty et al., 2024) and by comparing the density‐dependent Beverton‐Holt model to a density‐independent model. A quasi‐*R* ^2^ was calculated following Maceina and Pereira (2007). Model assumptions were assessed by visually inspecting residuals using the *nlstools* package.

## 3 RESULTS

### 3.1 Summary of *E*. *evelynae* abundance surveys

**FIGURE 2:**
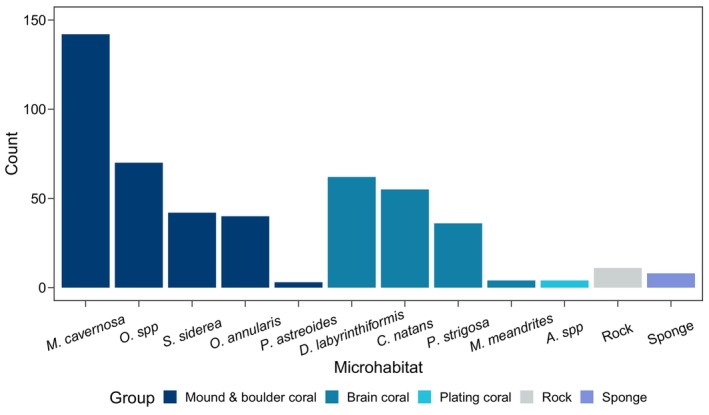
Bar plot of the number of observations for each microhabitat where *Elacatinus evelynae* was present across all sites. See Table S1 for a list of hosts (corals and sponges) with full scientific names.

We observed 1152 *E*. *evelynae* individuals across 61 transects. In total, we surveyed 478 microhabitats with gobies on them. Gobies predominantly occupied coral heads and were found on at least 10 coral taxa (Figure 2 and Table S1). We also rarely observed gobies on 11 rocks and eight sponges (four different species). Group size varied across microhabitats (mean ± SD = 2.41 ± 2.82 gobies per microhabitat). Considering all occupied microhabitats, 48% had a solitary goby and 23% had a pair; the largest observed group had 36 gobies.

### 3.2 *Elacatinus evelynae* preferentially associates with three coral host species

**FIGURE 3:**
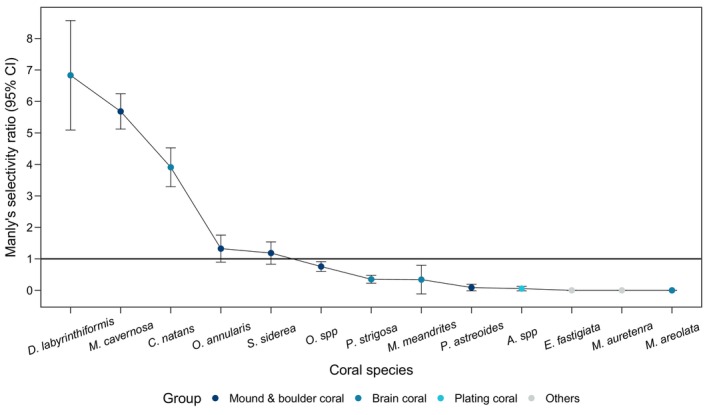
Manly's selectivity ratio for all observed coral species with 95% confidence intervals.

Of the coral species that the gobies were observed on, three species were strongly selected for—the brain corals *Diploria labrynthiformis* and *Colpophyllia natans*, and the boulder coral *Montastrea cavernosa* (Figure 3). The three preferred host species varied in size. *Colpohpyllia natans* had some of the largest coral heads observed (mean ± SD average diameter = 57.57 ± 19.15 cm), *M*. *cavernosa* had average‐sized coral heads (mean ± SD average diameter = 25.33 ± 10.62 cm), and *D*. *labrynthiformis* had small coral heads (mean ± SD average diameter = 19.38 ± 8.16 cm). *Elacatinus evelynae* did not preferentially inhabit the seven remaining coral host taxa, considering the corals' abundance on the reef. Three coral species, *Eusmilia fastigiata*, *Madracis auretenra*, and *Manicina areolata*, never hosted gobies despite being present in our study area (Figure 3).

### 3.3 Effect of microhabitat features on *E*. *evelynae* group size

At the microhabitat scale, coral species (χ^2^ = 89.71, df = 8, *p* < .001), average diameter (χ^2^ = 78.41, df = 1, *p* < .001), and health (χ^2^ = 20.45, df = 2, *p* < .001) were significant predictors of *E*. *evelynae* group size, whereas depth was not and was excluded from the final model (marginal *R* ^2^ = .46). Considering coral host species, the boulder coral *Montastrea cavernosa* hosted significantly larger groups than most other species, ranging from 3.0 to 7.8 times larger than groups on other corals, whereas the brain coral *Colpophyllia natans* hosted 5.2 times significantly larger groups than *Orbicella annularis* (Table 1). *Colpophyllia natans* also hosted marginally significantly larger groups than other *Orbicella* spp. (*p* = .097) and *Diploria labrynthiformis* (*p* = .084). On average, goby group size increased by 4.0% with every 1.0 cm increase in average coral diameter (incidence rate ratio = 1.04). Overall, healthy corals had significantly larger groups that were 1.6 and 2.9 times larger than intermediate and degraded corals, respectively (Table 1). Intermediate corals had 1.8 times larger groups than degraded corals, but this was marginally statistically insignificant (*p* = .063; Table 1).

**TABLE 1:** Pairwise contrasts from zero‐truncated negative binomial GLMM for coral species and health.

	Estimate (IRR)	S.E.	*Z*‐ratio	*p*‐value
**Coral species contrasts** ^a^				
*M*. *cavernosa* – *C*. *natans*	0.393 (1.482)	0.227	1.736	.724
*M*. *cavernosa* – *D*. *labrynthiformis*	**1.411 (4.100)**	0.291	4.856	**<.001**
*M*. *cavernosa* – *O*. *annularis*	**2.051 (7.772)**	0.342	6.003	**<.001**
*M*. *cavernosa* – *O*. spp.	**1.085 (2.958)**	0.199	5.444	**<.001**
*M*. *cavernosa* – *P*. *astreoides*	0.480 (1.616)	1.146	0.419	1.000
*M*. *cavernosa* – *P*. *strigosa*	**1.311 (3.708)**	0.298	4.395	**<.001**
*M*. *cavernosa* – *S*. *siderea*	**1.248 (3.485)**	0.264	4.727	**<.001**
*C*. *natans* – *D*. *labrynthiformis*	1.018 (2.767)	0.355	2.865	.097
*C*. *natans* – *O*. *annularis*	**1.657 (5.244)**	0.359	4.617	**<.001**
*C*. *natans* – *O*. spp.	0.691 (1.996)	0.237	2.921	.084
*C*. *natans* – *P*. *astreoides*	0.087 (1.090)	1.159	0.075	1.000
*C*. *natans* – *P*. *strigosa*	0.917 (2.502)	0.338	2.716	.142
*C*. *natans* – *S*. *siderea*	0.855 (2.351)	0.301	2.844	.103
*D*. *labrynthiformis* – *O*. *annularis*	0.639 (1.895)	0.436	1.467	.871
*D*. *labrynthiformis* – *O*. spp.	−0.327 (0.721)	0.331	−0.985	.987
*D*. *labrynthiformis* – *P*. *astreoides*	−0.931 (0.394)	1.176	−0.792	.997
*D*. *labrynthiformis* – *P*. *strigosa*	−0.101 (0.904)	0.392	−0.257	1.000
*D*. *labrynthiformis* – *S*. *siderea*	−0.163 (0.850)	0.374	−0.435	1.000
*O*. *annularis* – *O*. spp.	−0.966 (0.381)	0.356	−2.712	.143
*O*. *annularis* – *P*. *astreoides*	−1.571 (0.208)	1.192	−1.318	.926
*O*. *annularis* – *P*. *strigosa*	−0.740 (0.477)	0.432	−1.712	.739
*O*. *annularis* – *S*. *siderea*	−0.802 (0.448)	0.393	−2.040	.515
*O*. spp. – *P*. *astreoides*	−0.605 (0.546)	1.155	−0.524	1.000
*O*. spp. – *P*. *strigosa*	0.226 (1.253)	0.328	0.689	1.000
*O*. spp. – *S*. *siderea*	0.164 (1.178)	0.289	0.567	1.000
*P*. *astreoides* – *P*. *strigosa*	0.831 (2.295)	1.174	0.708	1.000
*P*. *astreoides* – *S*. *siderea*	0.769 (2.157)	1.165	0.708	.999
*P*. *strigosa* – *S*. *siderea*	−0.062 (0.940)	0.376	−0.165	1.000
**Health contrasts**				
Intermediate‐degraded	0.603 (1.828)	0.268	2.252	.063
Healthy‐degraded	**1.066 (2.903)**	0.267	3.990	<.001
Healthy‐intermediate	**0.462 (1.588)**	0.148	3.129	.005

*Note*: Incidence rate ratios are shown in parentheses after the estimates. Statistically significant estimates at α < .05 are bolded.

^a^ *Meandrina meandrites* excluded as it had very small estimates and SE of infinity due to small sample size.

When we restricted the dataset to only observations on the three preferred hosts (*M*. *cavernosa*, *C*. *natans*, and *D*. *labrynthiformis*) to assess interaction terms, we found the same main effects were significant (coral species, average diameter, and health), and there was a significant interaction between coral species and average diameter (Table S2). Post‐hoc tests revealed this interaction was driven by a stronger effect of average diameter in *M*. *cavernosa* compared with *C*. *natans* (Table S3). However, this model on the reduced dataset explained little variation in goby group size (marginal *R* ^2^ = .08), highlighting that the interaction term had little substantive effect, and that most of the explanatory power in the full model came from the species effect i.e., the larger group sizes on *M*. *cavernosa* and, to a lesser extent, *C*. *natans* relative to all other hosts.

### 3.4 Effect of coral cover on fish abundance at the 30‐m transect scale

**FIGURE 4:**
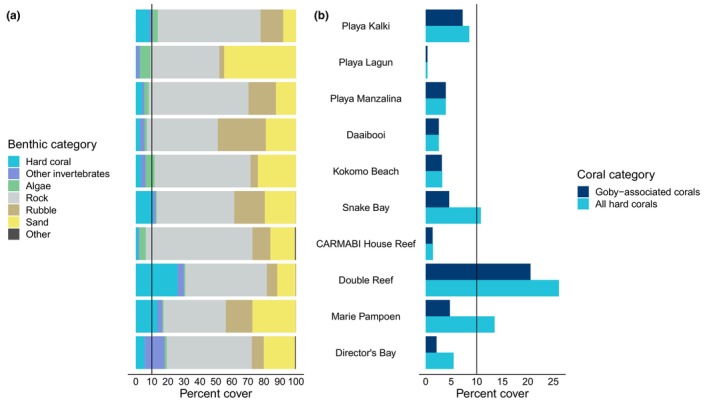
(a) Average benthic cover for all sites. (b) Cover of all coral species compared with cover of coral species that gobies can occupy (see list in Table S1). Sites are ordered geographically from West to East. The vertical line at 10% represents the minimum level of coral cover required to sustain a reef.

Estimated coral cover was low in most sites (Figure 4; mean ± SD = 7.52 ± 8.99%), falling below the 10% minimum threshold required for carbonate production and reef maintenance (Darling et al., 2019; Perry et al., 2013). Sites had significantly different average coral cover (Kruskal–Wallis Rank Sum Test: χ^2^ = 36.69, df = 9, *p* < .001; see pairwise Dunn Test in Table S4). The site with the highest level of coral cover was Double Reef with a coral cover of 26.10 ± 11.02% (mean ± SD), mainly comprised of two coral species occupied by gobies (*Pseudodiploria strigosa* and *C*. *natans*) and one species unoccupied by gobies (*Madracis auretenra*; Figure 4b). Marie Pampoen and Snake Bay also had average coral cover exceeding the 10% threshold. When considering only goby‐associated coral species, i.e., the percent cover of all coral species that *E*. *evelynae* was observed on (see Table S1), similar spatial patterns in coral cover were evident, although only Double Reef exceeded the 10% threshold. At the site level, these trends in coral cover qualitatively reflected the abundance of gobies in our surveys. Notably, Double Reef—the site with the highest average coral cover—also had the highest total goby abundance (*n* = 246 individuals and a mean ± SD = 41.0 ± 22.75 gobies per transect).

**FIGURE 5:**
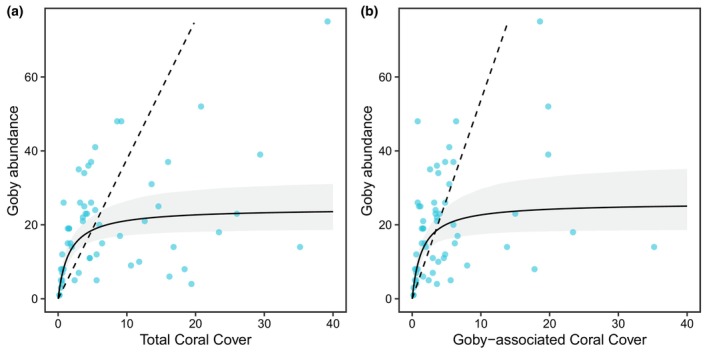
Beverton‐Holt curve for total goby abundance against (a) total cover of all hard corals, (b) cover of goby‐associated corals, with 95% confidence intervals. For total hard coral cover, a = 15.39 (95% CI: 10.15, 25.96) and b = 0.63 (95% CI: 0.35, 1.26). For goby‐associated coral cover, a = 18.00 (95% CI: 11.67, 31.67) and b = 0.69 (95% CI: 0.34, 1.54). Dashed lines represent a density‐independent curve.

There was, however, a weak relationship between *E*. *evelynae*'s abundance and coral cover at the transect scale (Figure 5). The fitted Beverton‐Holt curve modeled a relatively quick increase in goby abundance at low levels of coral cover, followed by an asymptote. However, we emphasize that there was substantial variation in the data around the fitted curve—notably, there were both small and large goby abundances at low coral cover, and some transects with high coral cover had goby abundances that exceeded the model's estimated asymptote. The model had a quasi‐*R* ^2^ of .24. For goby‐associated corals, the fitted curve for fish abundance exhibited a similar trend, with a quasi‐*R* ^2^ of .20. Residual inspection for both models confirmed an appropriate model fit, and both models were a better fit to the data than their respective density‐independent linear models (Figure 5).

## 4 DISCUSSION

As coral reefs undergo increased degradation, it is essential to understand how fishes will respond and at what spatial scale(s). In this study, we examined how *Elacatinus evelynae—*a functionally important cleaner fish*—*is impacted by habitat quality across multiple spatial scales. We found that at the microhabitat scale, coral species, size, and health were all important determinants of goby group size. Additionally, in terms of host specificity, we found that even though *E*. *evelynae* associates with at least 10 corals, it selectively inhabits just three coral species. Scaling up, we found that average coral cover tended to correspond with total surveyed goby abundance across distinct reef sites; however, there was a weak relationship between coral cover and goby abundance at the 30‐m transect scale. Below, we interpret our findings and discuss their implications in the context of future reef degradation.

### 4.1 Microhabitat quality affects *E*. *evelynae* group size

At the smallest spatial scale—the host microhabitat—both metrics of habitat quality (size and health of coral heads) were important determinants of *E*. *evelynae* group size, which varied from one to 36 individuals. Our finding aligns with previous work documenting a positive effect of coral size on *E*. *evelynae* group size (Ziebell et al., 2023). From a habitat constraint perspective, larger corals may simply provide more habitat space to accommodate larger groups of gobies, similar to other cryptobenthic fishes in which group size scales with microhabitat size (D'Aloia et al., 2011; Naaykens et al., 2024; Wong et al., 2005). Larger corals may also serve as a visual cue for settlers and clients (Igulu et al., 2011; Lecchini et al., 2014; Majoris et al., 2018), thereby attracting and maintaining more individuals. In addition to confirming the effect of host size, we provide new evidence that the host's approximate amount of live tissue (i.e., its health) is associated with *E*. *evelynae* group size. Put simply, healthier corals tended to host more gobies. Live coral tissue may be used as a visual cue by settlers, as documented in other reef fishes (Feary et al., 2007). Alternatively, resident *E*. *evelynae* may experience increased survival when inhabiting live coral. Several other fishes abandon degraded or dead coral hosts (Bonin et al., 2009; Feary et al., 2007; Pratchett et al., 2020), and recent monitoring work suggests that coral‐dwelling *Elacatinus* spp. may abandon diseased coral heads (Budd et al., 2024). However, the mechanisms underpinning this abandonment remain understudied. Possible explanations include increased rates of parasitism (Narvaez et al., 2021) and predation (Coker et al., 2009) for fish living on degraded corals. More research is needed to identify the fitness benefits *E*. *evelynae* receives from healthy coral hosts, particularly because Caribbean reefs are predicted to experience sustained degradation (Lawman et al., 2022; McManus et al., 2021).

Although our results underscore the importance of microhabitat quality metrics, our models could not fully explain variation in group size. Most strikingly, we found that gobies in Curaçao facultatively lived in groups, with nearly half of all microhabitats hosting a singleton, even though *E*. *evelynae* is predominantly described as living in breeding pairs or groups (Colin, 1975; Harding et al., 2003; Whiteman & Côté, 2003). This finding could be explained by individuals' post‐settlement movement patterns. For example, singletons observed on neighboring microhabitats may have been members of a breeding pair where the female is at the cleaning station and the male is guarding a nearby nest (Harding et al., 2003). Singletons may also be in the process of moving locally to search for a new host or a mate, though their post‐settlement movement capacities are limited (Harding et al., 2003; White et al., 2007). Alternatively, these individuals may be juveniles that happened to recently settle on unoccupied corals (note: we did not distinguish between juveniles and adults due to the challenges of size‐based classifications in situ). It is also possible that singletons were evicted from nearby groups. Whether group‐living *E*. *evelynae* form dominance hierarchies remains unknown, but the congener *E*. *prochilos* does so when occupying barrel sponges (Whiteman & Côté, 2004). Longer‐term tagging studies of all individuals within a neighborhood would uncover group stability and the frequency of small‐scale movements among hosts.

### 4.2 Implications of *E*. *evelynae's* host specificity

Despite occupying at least 10 coral species and, occasionally, sponges and rocks, *E*. *evelynae* selectively occupies just three corals in Curaçao. Two of the preferred host species—*M*. *cavernosa* and *C*. *natans*—also hosted the largest group sizes. A recent survey of coral‐associated fishes in Curaçao also documented large *E*. *evelynae* groups on these two species (Ziebell et al., 2023), highlighting their importance as goby hosts regionally. We speculate that the relatively large size of these corals (particularly historically) underpins this habitat preference. *Diploria labrynthiformis* was the third preferred host, but did not host large groups, likely because of the corals' small average size. *Diploria labrynthiformis* does, however, have deep grooves that offer a sheltered space that gobies often occupy. *Elactinus evelynae's* occupancy on at least seven other coral host species could be explained as (1) temporary stopover habitat during localized movements and/or (2) use of suboptimal habitat when their preferred hosts are scarce. Indeed, *Orbicella* spp. and *P*. *strigosa* were more abundant across our study area than all three goby‐preferred coral species, and *Agaricia* spp. and *Siderastrea siderea* were also more abundant than *C*. *natans* and *D*. *labrynthiformis* but were not significantly selected for by *E*. *evelynae*. Collectively, these results suggest that *E*. *evelynae* is not a specialist per se, as it occupies many microhabitats, but that it exhibits strong host preferences (Froehlich et al., 2023).

Having strong preferences for certain coral species could have negative implications for *E*. *evelynae*'s future survival in response to increased reef degradation and declining coral cover. The gobies' preferred coral species are among those most susceptible to stony coral tissue loss disease (SCTLD) (Alvarez‐Filip et al., 2019). As of the dates of our surveys, SCTLD had not yet been recorded in Curaçao (Kramer et al., 2022), however, in the summer of 2023, Curaçao witnessed its first outbreak of SCTLD. Surveys from the U.S. Virgin Islands during 2020–2021 revealed that *Elacatinus* spp. abundance declined on reefs where SCTLD was well‐established, compared with reefs where the disease was emerging or had not yet appeared (Budd et al., 2024). As disease outbreaks and bleaching events continue to occur, *E*. *evelynae* could be at risk of losing its habitat. Like other coral‐associated gobies, their long‐term persistence will partly depend on the flexibility of their habitat preferences, and their ability to form breeding pairs and/or groups on alternative host species (Froehlich et al., 2023).

### 4.3 Weak relationship between coral cover and *E*. *evelynae* abundance

Although total goby abundance was greatest at our highest coral cover site (Double Reef) and smallest on our lowest coral cover site (Playa Lagun), we documented a weak relationship between *E*. *evelynae* and coral cover along the surveyed 30‐m transects (quasi‐*R* ^2^ = .24). The observed pattern—inclusive of the strong variation around the fitted nonlinear curve—is analogous to empirical stock‐recruitment dynamics for which this class of nonlinear models was developed (Beverton & Holt, 1957). We note that at low coral cover, there is substantial variation in goby abundance (Figure 5). Thus, we speculate that at very low levels of coral cover, there is a risk that *E*. *evelynae* will decline in abundance and potentially be extirpated from the reef. Alternatively, an optimistic interpretation of these data is that *E*. *evelynae* could persist on relatively low coral‐cover reefs, so long as suitable host species are present.

Our results demonstrate that goby abundance cannot be explained by coral cover alone, and several factors could underlie this weak relationship. One possible explanation is that coral cover is a composite measure and, therefore, may not be a good predictor of ecosystem functioning or community interactions (Chan et al., 2023; Darling et al., 2013). In particular, coral cover does not capture community composition or the extent of homogenization (Chan et al., 2023). Also, coral cover may not inherently affect fish abundance, but, rather, may be a proxy for the structural complexity corals provide. Interestingly, even though *E*. *evelynae* exhibits strong preferences for certain coral host species, we fit a similar curve—which also had substantial variation around it—when we only considered the cover of corals that gobies can occupy in Curaçao (Figure 5b). However, we also acknowledge that other ecological factors, which we did not measure, will certainly impact local abundance. For example, as a cleaner fish, previous research has already shown that *E*. *evelynae* abundance is partly driven by clients (Cheney & Côté, 2003), and all fishes are influenced by spatial patterns of larval supply (Booth & Beretta, 2021; Doherty & Fowler, 1994; Shima, 2001).

The weak relationship between coral cover and goby abundance at the transect scale may also be influenced by the skew of our study sites toward low coral cover. We only surveyed three sites with relatively high average coral cover, defined here as >10%. Although we acknowledge that threshold is low, it is appropriate for Caribbean reefs, which were estimated to have an average coral cover of just 14–16% about a decade ago (Jackson et al., 2014; Schutte et al., 2010). Of those three sites, only Double Reef had high levels of corals that *E*. *evelynae* associates with (and, indeed, this site had, by far, the highest number of gobies). The other two “high” coral cover sites (Snake Bay and Marie Pampoen) were dominated by *Madracis auretenra*, a pencil coral that gobies do not inhabit. One region in Curaçao at the southeastern end of the island, Eastpunt, has higher coral cover (average: 25%, maximum: >40%) (CARMABI and Waitt Institute, 2017) but this area is not easily accessible, so we could not conduct surveys there. In the future, conducting more surveys in high coral cover sites would allow us to assess the fit of the saturation curve over a broader range of coral cover.

### 4.4 Conclusions and future directions

Collectively, our results demonstrate that habitat quality impacts *E*. *evelynae* abundance at multiple spatial scales. Given that Caribbean reefs are expected to experience further declines in live coral owing to repeated bleaching events (Lawman et al., 2022) and disease outbreaks (Alvarez‐Filip et al., 2022), fishes that specialize on coral hosts are susceptible to population declines and—potentially—extirpations. Although we found that *E*. *evelynae* selectively inhabits only three coral species and requires large, healthy corals to form groups, our data also offer some hope. Namely, we showed that gobies can sometimes persist on low coral cover reefs, and that they can at least occupy a broad range of hosts. Moving forward, it will be important to determine whether *E*. *evelynae* can establish cleaning stations and breeding grounds on new hosts if the local coral assemblage shifts. Against a backdrop of rapidly changing seascapes, the fate of *E*. *evelynae* and other coral dwellers may ultimately depend on the plasticity of their microhabitat affiliations.

## AUTHOR CONTRIBUTIONS

**Hana Fahim:** Conceptualization (supporting); formal analysis (lead); investigation (equal); validation (equal); visualization (lead); writing – original draft (lead); writing – review and editing (supporting). **Taylor Naaykens:** Conceptualization (supporting); investigation (equal); writing – review and editing (supporting). **Cassidy C. D'Aloia:** Conceptualization (lead); formal analysis (supporting); funding acquisition (lead); investigation (equal); resources (lead); supervision (lead); validation (equal); writing – original draft (supporting); writing – review and editing (lead).

## CONFLICT OF INTEREST STATEMENT

The authors declare that there are no conflicts of interest.

## Supporting information


Table S1‐S4:

## Data Availability

The data and code that support the findings of this study are available on Dryad: https://doi.org/10.5061/dryad.z8w9ghxmg.
