# Supplementary material for: Habitat quality effects on the abundance of a coral‐dwelling fish across spatial scales
Source: Ecol Evol. 2024 Sep 22;14(9):e70322. doi: 10.1002/ece3.70322 (PMC11416863; doi:10.1002/ece3.70322)
Supplement: Supplementary file 1 — Table S1‐S4: [file ECE3-14-e70322-s001.pdf]

Supplementary Information:

Habitat quality affects the abundance of a coral-dwelling fish across spatial scales

Hana Fahim[1,2], Taylor Naaykens[1,2], Cassidy C. D'Aloia[1,2*]

[1] Department of Biology, University of Toronto Mississauga, Mississauga, L5L 1C6, ON, Canada

[2]Department of Ecology and Evolutionary Biology, University of Toronto, Toronto, M5S 1A1, ON, Canada

*** Corresponding author contact:**
Cassidy D'Aloia (cassidy.daloia@utoronto.ca)
Address: Department of Biology, University of Toronto Mississauga, 3359 Mississauga Road, Mississauga, ON, L5L 1C6, Canada

Table S1. List of all host species *E. evelynae* was observed on.

Species scientific name	Species common name
**Mound & boulder corals**	
Montastraea cavernosa	Great star coral
Orbicella annularis	Boulder star coral
Orbicella spp. (O. franksi & O. faveolata)	Star coral & Mountainous star coral
Porites astreoides	Mustard hill coral
Siderastrea siderea	Massive starlet coral
**Brain corals**	
Colpophyllia natans	Boulder brain coral
Diploria labrynthiformis	Grooved brain coral
Meandrina meandrites	Maze coral
Pseudodiploria strigosa	Symmetrical brain coral
**Plating corals**	
Agaricia spp. (A. agaricites & A. lamarcki)	Lettuce & sheet coral
**Sponges**	
Aplysina archeri	Stove-pipe sponge
Ircinia campana	Vase sponge
Ircinia sp.	Unknown
Neofibularia nolitangere	Touch-me-not sponge

Table S2. Type II Wald Chi-squared Test of fixed effects for zero truncated negative binomial model explaining goby group size that includes an interaction term between host species and average diameter. This model is based on observations using only the three preferred hosts (*M. cavernosa, C. natans,* and *D. labrynthiformis*) and explains little variation in the response variable (marginal $R^2 = 0.08$).

**Variable**	χ^2	**d.f.**	**p-value**
Average diameter	76.14	1	**<.001**
Host species	23.20	2	**<.001**
Condition	30.98	2	**<.001**
Average diameter × host species	10.99	2	**.004**

Table S3. Pairwise slope contrasts for the interaction between coral host species and average diameter from the "emtrends" function for the model presented in Table S2. Results averaged over levels of "condition" and p-values are Tukey-adjusted.

**Contrast**	**Estimate**	**S.E.**	**Z-Ratio**	**p-value**
M. cavernosa – C. natans	0.039	0.012	3.315	**.003**
M. cavernosa – D. labrynthiformis	0.018	0.028	0.657	.788
C. natans – D. labrynthiformis	-0.021	0.028	-0.747	.736

Table S4. List of coefficients from Dunn test for site differences in coral cover.

Coefficient	Estimate	p-value
CARMABI House Reef - Daaibooi	-0.701	0.242
**CARMABI House Reef - Director's Bay**	**-1.788**	**0.037**
**CARMABI House Reef - Double Reef**	**-4.425**	**0.000**
**CARMABI House Reef - Playa Kalki**	**-2.019**	**0.022**
CARMABI House Reef - Kokomo Beach	-1.244	0.107
CARMABI House Reef - Playa Lagun	0.643	0.260
CARMABI House Reef - Playa Manzalina	-1.565	0.059
**CARMABI House Reef - Marie Pampoen**	**-3.276**	**0.001**
**CARMABI House Reef - Snake Bay**	**-3.285**	**0.001**
Daaibooi - Director's Bay	-0.992	0.161
**Daaibooi - Double Reef**	**-3.400**	**0.000**
Daaibooi - Playa Kalki	-1.204	0.114
Daaibooi - Kokomo Beach	-0.496	0.310
Daaibooi - Playa Lagun	1.171	0.121
Daaibooi - Playa Manzalina	-0.789	0.215
**Daaibooi - Marie Pampoen**	**-2.350**	**0.009**
**Daaibooi - Snake Bay**	**-2.359**	**0.009**
**Director's Bay - Double Reef**	**-2.407**	**0.008**
Director's Bay - Playa Kalki	-0.211	0.416
Director's Bay - Kokomo Beach	0.496	0.310
**Director's Bay - Playa Lagun**	**2.059**	**0.020**
Director's Bay - Playa Manzalina	0.203	0.419
Director's Bay - Marie Pampoen	-1.358	0.087
Director's Bay - Snake Bay	-1.366	0.086
**Double Reef - Playa Kalki**	**2.196**	**0.014**
**Double Reef - Kokomo Beach**	**2.903**	**0.002**
**Double Reef - Playa Lagun**	**4.212**	**0.000**
**Double Reef - Playa Manzalina**	**2.611**	**0.005**
Double Reef - Marie Pampoen	1.049	0.147
Double Reef - Snake Bay	1.041	0.149
Kokomo Beach - Playa Lagun	1.615	0.053
Kokomo Beach - Playa Manzalina	-0.293	0.385
**Kokomo Beach - Marie Pampoen**	**-1.854**	**0.032**
**Kokomo Beach - Snake Bay**	**-1.862**	**0.031**
Marie Pampoen - Snake Bay	-0.008	0.497
Playa Kalki - Kokomo Beach	0.708	0.240
**Playa Kalki - Playa Lagun**	**2.248**	**0.012**
Playa Kalki - Playa Manzalina	0.415	0.339
Playa Kalki - Marie Pampoen	-1.147	0.126
Playa Kalki - Snake Bay	-1.155	0.124
**Playa Lagun - Playa Manzalina**	**-1.877**	**0.030**
**Playa Lagun - Marie Pampoen**	**-3.273**	**0.001**
**Playa Lagun - Snake Bay**	**-3.281**	**0.001**
Playa Manzalina - Marie Pampoen	-1.562	0.059
Playa Manzalina - Snake Bay	-1.570	0.058

Note:
Statistically significant estimates are highlighted in bold, using $\alpha = 0.05$
